# Prevalence, Associated Factors, Barriers and Facilitators for Oral HIV Self-Testing among Partners of Pregnant Women Attending Antenatal Care Clinics in Wakiso, Uganda

**DOI:** 10.21203/rs.3.rs-4378165/v1

**Published:** 2024-05-15

**Authors:** Lawrence Nduhukyire, Fred C. Semitala, Juliet Ntuulo Mutanda, Dan Muramuzi, Patrick Albert Ipola, Allen Kabagenyi, Joan Nangendo, Juliana Namutundu

**Affiliations:** Makerere University; Makerere University; Makerere University; Makerere University; Makerere University; Makerere University; Makerere University; Makerere University

**Keywords:** Oral HIV Self-Testing, Partners

## Abstract

**Background:**

Oral HIV self-testing (HIVST) among men is relatively low and still inadequate in Sub-Saharan Africa. Delivering HIVST kits by pregnant women attending antenatal care to their partners is a promising strategy for increasing HIV testing among men. However, even amidst the interventions, most men do not know their HIV status. This study, aimed to determine the proportion of partners who received and used oral HIVST kits delivered by pregnant women, associated factors, barriers, and facilitators for uptake among partners.

**Methods:**

We conducted an exploratory sequential mixed methods study among 380 sampled partners. Lists of partners in the HIVST log books whose women picked an HIVST kit were obtained and systematic random sampling was done to obtain participants. 14 male partners were purposively selected for in-depth interviews (IDIs) to identify barriers and facilitators. We used modified poison regression to determine the association between oral HIVST and independent variables. We used an inductive analysis for the qualitative analysis.

**Results:**

Out of 380 participants, 260(68.4%) received an oral HIVST kit from their pregnant women, and 215(82.7%) used the kit for HIVST. Oral HIVST was associated with; Information Education and Communication (CPR = 1.64, 95%CI 1.48–1.82), being reached at home (CPR = 1.04, 95%CI 1.01–1.08), and being aware of the woman’s HIV status (CPR = 1.04, 95%CI 0.99–1.09). In-depth results identified barriers to uptake as, lack of trust in the HIVST kit results, fear of test outcome in the presence of the partner and inclination that the HIV status of their women is the same as theirs, and facilitators included convenience, ease to use, prior awareness of their HIV status, and fear of relationship consequences and breakup.

**Conclusion:**

Delivery of oral HIVST kits to men through pregnant women reached a high number of men and achieved a high uptake. Accessing information, education, communication and convenience nature were the major reasons for uptake among men who received the Oral HIVST kit as trust issues of the kit affected use among partners. Scaling up the delivery of oral HIVST kits at all departments of the hospital through women seeking health services is paramount to support HIV screening among men to reach the UNAIDS 95 strategy.

## Background

Globally, HIV is a public health burden, an estimated 40 million people were living with HIV in 2023, an estimated 1.7 million became newly infected with HIV, and 880,000 deaths occurred around the globe [1]. Approximately 5.5 million people were not aware that they had HIV and still needed access to HIV testing services [1], as sub-Saharan Africa (SSA) remains the most affected with more than 75% of which 45.7% of newly diagnosed cases being among adults [2]. The Joint United Nations Programme (UNAIDS) set a 95-95-95 target to be reached by 2030 [3]; meaning that the global target for HIV status awareness is 95% where reaching this cascade requires having more people tested for HIV. The results of the 2020 Uganda Population-based HIV Impact Assessment (UPHIA) indicate that the current prevalence of HIV among adults (15 to 49) years in Uganda is 5.5% [4]; thus, with this prevalence, increasing access to HIV testing and early diagnosis is crucial for controlling further spread of the disease.

There are various approaches used for testing HIV but for many settings, the World Health Organisation (WHO) recommends the use of rapid diagnostic tests (RDTs) to accelerate the uptake of HIV testing since they allow quicker provision of test results. With appropriate training, education (Information, Education and communication), support, and supervision, community health workers can use RDTs to perform HIV testing with accuracy and reliability [5]. In Uganda, HIVST has gained significant attention after being implemented and enrolled by the Ministry of Health in ANC clinics as one of the ways to have men tested for HIV [6, 7].

Determining the factors associated with oral HIV self-testing among men is essential for identifying categories that need targeted interventions and closing gaps in HIV testing coverage for this group. Implementation of HIVST in Uganda supported by MOH [6, 7] showed high acceptability and use of HIVST by partners of pregnant women. However, there remain key concerns by policymakers worldwide and Uganda about HIV self-testing including; lack of policies and regulatory systems, quality of oral HIV self-test kits, ethical and human rights issues, lack of counselling that may increase suicidal cases and knowledge about HIVST kits before use [8, 9]. Where-as there is a rationale for improving the uptake of oral HIVST services and subsequently HIV testing as a gateway to accessing lifesaving ART and prevention strategies, results from studies that informed enrollment of the HIVST kits in ANC setting in Uganda revealed uptake of HIVST was moderate at 76% [6, 7]. However, there is limited evidence-based research that has been carried out among men to evaluate the intervention since its implementation. Evidence about its use by partners of pregnant women is important to inform necessary improvements and this study timely to assess the actual delivery and uptake since there has not been any evaluation done since the intervention was rolled out Most studies targeted pregnant women as a proxy measure for uptake of HIVST among their male partners. Thus, our study focused on male partners themselves to ascertain those who received and used the oral HIVST kits. Therefore, this study determined the actual delivery of HIVST kits, uptake by the partners who received the kits, and the factors associated, barriers and facilitators for use of HIVST kits among partners of pregnant women attending Antenatal clinics in Uganda.

## Methods

### Study Design

A cross-sectional survey was conducted using a sequential explanatory mixed methods design approach with the partners of Pregnant women attending ANC at a public health facility. The quantitative component involved collecting information using an electronically designed questionnaire from male partners, face to face with participants to determine the proportion that received an oral HIVST kit from their ANC-attending women; and partners that used it for HIV self-testing, and associated factors for uptake of oral HIVST. The qualitative component used in-depth interviews (IDI) to identify the facilitators and barriers to uptake of kits delivered by pregnant women. The questionnaire was pretested before data collection by trained research assistants in ethics who held at least a bachelor’s degree

### Study Setting

The study was carried out at Wakiso Health Centre IV, a government health facility in Wakiso district. The (HCIV) provides free ANC services to women serving up to 250 or above women daily. Wakiso district houses most of the residents working in Uganda’s capital city and business centre, Kampala; with men who are often busy working, and unable to escort their women for ANC–a point entry where men could have an opportunity to be screened for HIV. The district is located in Central Uganda, a region with the highest HIV prevalence at 8.1% above the national adult prevalence of 5.8% [10].

### Study Population

The study population were partners of pregnant women who were attending ANC at Wakiso HCIV in Wakiso District. Male partners were targeted because the MoH rolled out HIVST at ANC clinics to have the partners of pregnant women tested for HIV at their convenience since they often had no time to accompany their pregnant women to ANC where they would have an opportunity to be tested and to minimize the spread of HIV infections among mothers and the un born babies [11, 12]. The study excluded male partners whose telephone number was not provided in the HIVST distribution log book at the ANC by their pregnant woman before receiving the kit.

### Sampling and sample size estimation

The sample size was determined based on the Kish Leslie (1965) formula n = Za^2^ p(1-p) / σ^2^, with (n) for the study sample size for male partners whose women attended the antenatal clinic, Za Standard normal deviate at 95% confidence interval (CI) corresponding to 1.96, P for the prevalence of HIV testing among men which was assumed at 67% [13], 1 – P for Probability of Partner’s use of oral HIVST, and 0.05 as margin of error.

Study participants were selected using a systematic random sampling technique from the target population of ANC pregnant women visiting the ANC clinic. A total number of 380 partners to pregnant women who agreed to deliver HIVST kits were considered to participate in the study. This sample size estimation ensured that the study would have sufficient statistical power to estimate the population prevalence of uptake among partners of pregnant women in Antenatal clinics in Uganda considering a 95% CI. To ascertain the number of male participants, the HIVST distribution log at the ANC clinic was reviewed and a list of all pregnant women who picked the kits for their male partners drawn from January 2021 to October 2022 was chosen and used as the study sampling frame for selection. After attaining the lists, the total number of pregnant women who accepted to deliver oral HIVST kits to their partners was the numerator and the total sample size was a denominator to attain the sampling interval (n^th^) number during the recruitment of participants. The male participants were obtained using the n^th^ number and where every n^th^ fell, and a male partner’s phone number was provided, was considered eligible to participate in the study.

### Study Procedure

Individual male partners eligible were contacted to schedule an interview with them and were willing to consent before participating in the study. During the phone call to schedule an interview, we introduced to the male partner the purpose of the study as; we are looking at every mother who received ANC from our facility Wakiso HCIV. For male partners who were not willing to come to the study site for the face-to-face interviews, we scheduled to visit them in their community and collected data from them at their convenience areas. This is because men are rare and any chance, they are ready to provide information could not be missed

Qualitative data was collected by trained research assistants using an in-depth interview guide. The IDIs were conducted with the partners of pregnant women who picked oral HIV self-test kits to deliver to their male partners for HIV testing at home and registered a valid partner’s telephone number in the HIVST distribution log. An exploration of barriers limiting the use of kits among men delivered by their pregnant partners was studied. Questions asked to male partners of pregnant women were about the process of how the HIVST kit was delivered to them, what their pregnant women told them, the process of testing, actions after testing, what motivated them to use the HIVST kit and barriers related to none use of the oral HIVST kit. This helped to record the views of the men themselves on the barriers affecting uptake and reasons for the use of kits. All discussions and interviews were audio-recorded with permission from the participants. IDI lasted between 30–40 minutes.

### Measures

The main outcome of interest was; (i) Receiving the oral HIVST kit (ii) Using the oral HIV self-testing kit. The primary outcome variable was measured with a set of questions asked from partners of pregnant women to determine those who received and used the HIVST. The questions were asked in a way that did not directly involve the pregnant women and this was done to reduce the risk of increasing domestic violence in homes **1**) Are you aware of your HIV status? **2**) when did you last test? **3**) have you ever received an HIVST kit? **4**) From whom did you receive the HIVST kit? **5**) Did you use the received HIV self-testing kit to test yourself for HIV? **6**) Were you able to obtain results after using the HIVST kit? Uptake of the oral HIVST was measured as a binary outcome if the participant responded “Yes” to the question.

### Data analysis

Quantitative data analysis was conducted using Stata-version 14. Analysis was conducted at three levels; univariable, bivariable, and multivariable analysis. In univariate analysis, the mean (standard deviation) of the age of respondents; frequencies and percentages of the independent variables were reported. Frequencies and percentages were calculated for descriptive statistics including the number of partners that received the HIVST kits and the prevalence that used the kits for HIV self-testing. To determine the factors associated with the uptake of oral HIVST, modified Poisson regression was used to estimate prevalent ratios (PRs) rather than odds ratios since the outcome prevalence exceeded 10% [14].

Originally, unadjusted PRs were generated to select candidate variables for inclusion in the multivariable model where variables with a p-value (P < 0.25) at bivariate analysis. Variables found significantly associated with the outcome in literature were also considered potential for the multivariable model. Multicollinearity analysis was performed but no variable was found to be linear to another thus none was removed at (p < 0.40).

Variables selected above were entered into a multivariable modified Poisson model to determine adjusted PRs. A stepwise procedure involving a forward and backward elimination approach was used to select variables after considering significant variables at p < 0.05. This modelling strategy determined independent predictors of uptake of oral HIVST by partners to pregnant women attending ANC after controlling for confounding variables. A goodness of fit test for the modified Poisson model was checked by considering the AIC values. The model with the smallest AIC figure was considered. The Poisson model allowed the estimation of PRs compared to odds ratios that would overestimate the associations given the high prevalence of the outcome.

Qualitatively; All audio-recorded verbatim were transcribed by experienced research assistants who held a Bachelor’s degree, were fluent in Luganda and translated the audio interviews into English for analysis. The translated soft copies were printed into hard copies for review by the PI and two other independent qualitative researchers to assess accuracy and completeness. This helped to familiarize me with the transcripts before analysis. All transcripts were imported into Dedoose software for analysis to identify codes and themes based on the primary objective of barriers and facilitators to oral HIVST among partners of pregnant women who received HIVST kits from the clinic. All emerging subthemes about barriers and facilitators applied initial codes for inductive thematic analysis. Extraction of key quotations, explanations, and interpretations from the analysis was done.

## Results

### Socio-Demographic Characteristics of Participants

We enrolled 380 participants with a mean age of 30.1 years (SD 6.2), 53.9% (205) were married, 54.5% (207) had at least one sexual partner besides their spouse and the majority 66.9% (252) had secondary education (Table 1).

### Proportion of partners to ANC women who received oral HIV self-testing kits

**Figure 2:**
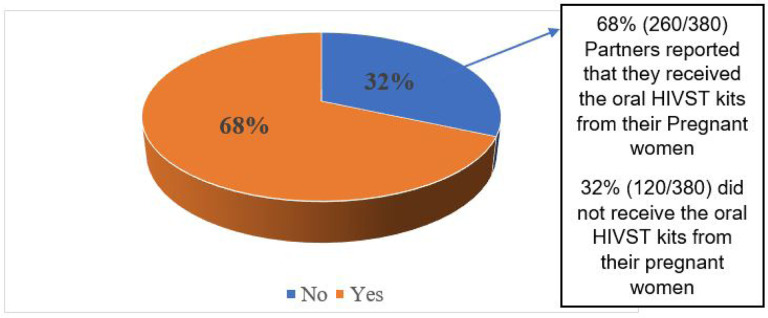
Proportion of Male partners who received an HIVST kit from their pregnant partners

Over half (68.4%; 260/380) of pregnant women who picked oral HIVST kits delivered them to their partners for HIV self-testing (Fig. 2).

### Uptake of HIV self-testing kits

Self-reported uptake of oral HIVST kits by the male partners was 82.7% (215/260) (Fig. 2)

#### Factors associated with uptake of oral HIV self-testing among partners of pregnant attending antenatal care.

At bivariate analysis, marital status, staying with a partner, information education and communication, pre-HIV phone counselling, place of self-testing, spouse’s level of education, being aware of the spouses’ HIV status and perceived risk of contracting HIV were the factors associated with uptake of oral HIV self-testing. (Table 2)

At multivariable analysis, we found that uptake of oral HIVST was significantly associated with having access to information education and communication about HIVST (aPR: 1.64; 95%CI: 1.48–1.82); being aware of a partner’s HIV status (aPR: 1.04; 95%CI: 0.99–1.09); and being reached at home (aPR: 1.04; 95%CI: 1.01–1.08) Table 2.

#### Facilitators for oral HIV self-testing among partners of pregnant women who received the kit attending ANC at Wakiso HCIV

Among the facilitators for oral HIVST was partner testing because this helped the two know each other’s status. Men revealed that they tested for HIV in the presence of their pregnant women which promoted partner testing. Partners easily understood each other’s status paving way for keeping safe when they test together.

……. Yes, she was there, after swabbing, she picked it from me and put it in the buffer, and showed me the control test”

[IDI, P002U, 25years]

…….. *we tested very well together, it was 7 pm, and that day I went home early, I tested at about 7:30 minutes and when each of us found that we were safe, each of us slept very well when we understood that each of us was HIV Negative”*

[IDI, P001, 31years]

### HIVST is a convenient, easy, and harmless way of HIV testing

Men revealed that there was no waste of time moving to facilities since the kits were delivered to them at home and did not involve piercing to test for them themselves. One of the respondents reported that he was able to test for HIV because of the convenience of the HIVST kit delivered by his wife.

“ ..…I didn’t have time because when the time approached to come there, she then told me they wanted us to go together for ANC then I said no because my job can’t allow me to go there so, then she told me now, they have given me this, it is the thing to test HIV, I said it is okay, I can use it no problem…”

[IDI, P010U, 28years]

Men identified that using the kit was easy because of the instructions. Some noted that because they did not need to be pierced before being tested was an automatic yes for them to use the kit when their pregnant women brought it home.

…..I haven’t seen any barriers concerning the use of the HIVST kit, in my own point of view, it is actually very convenient and good, instructions are understandable, easy to use, and they don’t prick you to obtain blood for the test; it’s painless, doesn’t vibrate, you don’t have any fear of wounds”

[IDI, P012U, 31years]

#### Being aware of their HIV status before using the oral HIV self-testing kit as a facilitator for use of the kit

Male partners who knew their HIV status easily used the HIVST kit delivered by their pregnant partners. A respondent reported that using the HIVST kit was not a problem since he had tested and knew his status before using the kit their partner delivered

……When my wife brought the kit, I did not use it immediately because I was not sure of my before so I went to a clinic, when the nurse checked me, I got the results, and I was negative She charged me 7000 shillings as service. When I reached home, I asked for the one my wife had brought and used it too

[IDI, P012U, 31years]

### Fear of Negative Relationship Consequences

Some men revealed that they used the oral HIVST kit due to the fear they developed related to losing their women if they did not use the kit, pregnant women brought home.

……She threatened me to leave **and**, I didn’t want to lose her and for the safety and health of our baby, I had to use the thing she has brought.

[IDI, P002U, 25years]

### Barriers to the Use of HIV self-testing kits by male partners Concerns over the accuracy of the kit and fear of obtaining incorrect results

Participants raised concerns over the accuracy of kits and obtaining incorrect results that could cause violence. They revealed that however willing they would be to use the kits, they were uncomfortable with the results.

……. Personally, I still doubt how genuine the results turn out because what I know is; HIV is transmitted through blood, even if you give me 100, it’s hard for me…”

[IDI, P003U, 27years]

Male partners lacked the confidence to use the HIVST kits due to a lack of education and limited access to information about the kits that were availed to them, and their functionality.

…….. I had a lot of pressure thinking I would not get the right results reason being that I had one friend of mine who went to a facility to test for HIV and he was told he had HIV and when he later tested again from another facility, he was told he was negative So that pressure of false results really discourages me from using the oral kit to test my-self. so, once I know my wife’s results, I can also know how my status is hahaha.

[IDI, P012U, 31years]

However, some respondents also raised concerns about how one could control himself in case he tested positive using the kit being that testing was done in the absence of trained personal.

…….. What if a person used it and got positive results? Usually, such a person is given counseling. However, in such a scenario, you’ve given it [testing kit] to this person, when he has not asked for it. He does the test from his home and gets results yet there isn’t any counseling. What could happen next to such a person? How can you help such a person when you are not there with him?

[P011, 31years]

### The male superiority complex and inclination that women’s HIV status is the same as theirs

Male partners who did not use the delivered HIVST kits expressed feelings that defined powers either or not to use the kits unless they wanted to do so. In Uganda, men are heads of the family and are respected by their women and children. They are decision-makers thus if they refused to use the kit nothing would go wrong

……. In the first place, I was not consulted that my wife was going to deliver an HIV testing material, this alone was a demotivation for me to use the testing kits she brought. If I used it like easily, that means she would start forcing me to do other things she felt she wanted”

[IDI, P006, 35years]

Some male partners also revealed that due to the stigma related to HIV testing, they consider themselves to have the same HIV status similar to that of their female partners. They claimed that if their woman tested for HIV, they did not need to retest for HIV as they considered the outcome of females as theirs too.

……. Most times I take my spouse’s results, once I know her results, then I can conclude what my results could be…”

[IDI, P012U, 31years]

## Discussion

The study determined the actual delivery and uptake of oral HIVST and associated factors, barriers and facilitators for the use among partners of pregnant women attending Antenatal care clinics in Wakiso, Uganda. We found that 68.4%partners received the oral HIVST kits from their ANC women attending antenatal care at a public health facility for HIV testing and uptake of a service was at 82.7%. The variable factors associated with uptake were; having access to information education and communication about HIVST, being aware of a partner’s HIV status, and being reached at home. This indicates that the strategy of delivering HIVST kits through pregnant women attending antenatal care has the potential to reach a significant portion of men who may not have otherwise accessed HIV testing services.

Besides, our findings indicated that not all ANC women who picked an oral HIVST kit to deliver to their partners delivered it to the partner hence this limits the cascading of the HIV testing strategy. This percentage distributed is lower compared to a study which found that 91% of ANC attendees reported successfully distributing HIVST kits to their male partners, which facilitated couples testing [15]. The percentage of women who did not deliver the oral HIV kits was relatively high given the purpose of the intervention which intends to reach all men of ANC attending women. Irregular communication among partners are some of the challenges for the non-delivery of the kits to the male partners [16], and some women never deliver the HIVST kits due to fear of their partners’ reactions [16]. Our study revealed that some women used the oral HIVST kits on other family members and by the time the partner came back home, the oral HIVST kit was already used, other women had misplaced the kits and did not know where the kits were among other reasons. These results suggest the need to strengthen strategies that will ensure women deliver oral HIV self-testing kits to the intended population. Studies can be done to explore why women pick the kits to deliver them but end up not giving them to the intended persons.

The uptake of HIVST among partners of pregnant women was 82.7%. Results from the study are slightly higher than those of a randomized trial whose uptake was 76% [7]. The high uptake in this study is a significant achievement in addressing the longstanding challenge of low HIV testing rates among men, particularly in Uganda and sub-Saharan Africa. The results indicate that once men have access to the kits, they are generally willing to self-test for HIV. However results from our study are slightly lower than those from similar studies whose uptake was high at 86% and 97.7% respectively [17, 18]; but higher than that of the study among men in Zambia whose uptake was at 24% respectively [19, 20].

This may be attributed to the convenience and accessibility; the trust and influence women hold as they are perceived to be good caregivers and knowledgeable individuals regarding health matters in families. This also shows that the confidentiality created through this unique delivery approach has collectively contributed to a high number of male partners accessing HIV testing services using the oral HIVST kits delivered by their pregnant partners at home.

The modified factors that were associated with the uptake of oral HIV self-testing among partners of pregnant women were information, education, and communication (IEC), place of HIV self-testing, and being aware of the woman’s HIV status. Access to IEC and materials helped partners easily understand how to use the oral HIVST kits. The association between oral HIVST and IEC has been well-documented in previous studies [21, 22]. This highlights the importance of effective communication and education campaigns through media like televisions, social media, radios, and health education targeted at promoting awareness and knowledge about HIVST to empower individuals with the necessary information to make informed decisions.

From the in-depth interviews, male partners attributed the smooth explanation of how to use the HIVST kits to the education level of their pregnant partners. It is possible that women with a high education level were able to explain the processes of HIV testing as directed by the health workers. These findings suggest that comprehensive education campaigns provide accurate information and address potential concerns surrounding HIVST. These campaigns can help increase awareness and knowledge of oral HIVST especially among this group. This is in line with other studies [16, 23], which recommend the need to intensify health education and communication through various media oral HIVST, as this approach is to reach more men and convince them to test for HIV using the delivered HIVST kits by their pregnant women. The gaps in the IEC strategy about the HIVST include limited access to oral HIVST information, affecting participants to perform self-testing due to a lack of knowledge and awareness on how to use the kit. There is a need to intensify awareness about HIVST use and education on its use among the communities to ensure the secondary delivery of kits yields the best results intended ultimately contributing to better prevention and early detection of HIV in this population.

Our findings indicated that more male partners tested when reached at home. The preference for delivered oral HIV self-testing, as demonstrated in this study, is in line with existing evidence [24, 25]. The findings of this study indicate that the secondary delivery of HIVST kits could improve male partner HIV testing due to the convenience and time issues. The flexibility of this model addresses barriers associated with facility-based testing, such as stigma and time constraints. Scaling up the secondary delivery of oral kits to all departments of the hospitals into a routine distribution through women attending all departments could help address the low testing rate currently in this population. [26, 27]. These results suggest that by making testing more accessible, secondary delivery of oral HIVST can potentially reach a larger population and improve overall testing rates.

In addition, the convenience and ease of use was a major facilitator of oral HIVST use. Participants expressed appreciation for the simplicity and accessibility of the self-testing kits through their women without necessarily moving to the facility themselves. This has also been highlighted in previous studies [28–30], because the secondary delivery of oral HIVST kits addresses barriers such as transportation costs and time constraints, enabling men to test for HIV at their convenience. However, important to note was that fear of relationship consequences facilitated use of the kit according to in-depth interviews Some men highlighted concerns about potential negative impacts on their relationships if they did not use the HIVST kit that was delivered by their pregnant partners. This forced this category of men to test so that they could save their relationships from being broken.

This however echoes stigma in the process. Previous studies have found similar reasons as barriers to oral HIVST among men [31–33]. However, our study findings showed that Prior awareness of one’s HIV Status encouraged male partners to do an HIV Self-test. This suggests that oral self-testing serves as a means of regular monitoring for individuals already aware or even engaged in HIV care/prevention programs. Similar findings were reported in a previous study [15].

More interestingly, most participants revealed that they were motivated to use the HIVST kit because helped them understand each other’s HIV status as a couple. This emerged as a significant motivation oral self-testing by male partners. Similar findings are in line with other research carried out [34]. These findings indicate that testing together as partners allows for shared responsibility, open communication, and support in maintaining a healthy relationship.

While oral HIVST was found to be convenient and used by the majority, a common view among male partners during in-depth for failure to use the kit was due to concerns and fear of obtaining incorrect results. Participants expressed concerns about the accuracy of HIVST kits and the fear of obtaining incorrect results in presence of their partners. This fear could have raised from concerns about potential consequences within the relationship, being stigmatized, blamed, or even bringing about violence. This is a well-known barrier according to previous studies that highlighted the psychological issues related to obtaining inaccurate results [17, 35–37]. Given the study, there is need to address these concerns through awareness campaigns, information, educational and communication materials, with clear instructions on kit usage and interpreting results. This can help eliminate doubts, ensure confidence, be user friendly, trust and encourage uptake of oral HIV self-testing by this population. Promoting pre/post-HIVST partner counseling either through phone, or confidentiality, can help address such concerns among this population.

Interestingly, some of the participants revealed that they did not need to test for HIV because their partner’s status is the same as theirs. Participants had an assumption that a woman’s HIV status is the same as theirs, leading to a decreased perceived need for the individual using the delivered kit to test themselves. Similar findings were reported in a previous [38]. Such misconceptions likely are caused by a lack of communication and understanding about the dynamics of HIV transmission. Given the findings, providing accurate information about HIV transmission, the importance of individual testing, and the potential for serodiscordant relationships can help demystify the misconception and encourage individuals to carry out HIV testing. Pregnant women attending ANC are given one HIVST kit for their partners hence if the kit is misplaced, partners miss the opportunity of being tested. Participants during in-depth interviews mentioned instances where the kit was misplaced, used by someone else, or not readily available having been used by another family member. This logistical barrier can be addressed through improved distribution strategies, ensuring sufficient availability promoting proper storage and safeguarding kits within households when given for delivery. Leveraging these strategies is feasible in a resource-limited setting like Uganda provides a major opportunity to strengthen and cascade HIV screening an entry to HIV care and prevention among this population.

The study had some limitations. Uptake of HIVST was measured by self-reports from male partners and were unfortunately not confirmed elsewhere in the study. This could have possibly allowed misreporting and may also have been subject to desirable responses.

Another limitation was, to better understand the differences in men who received the test kits, it could have been important to explore the demographic and contextual factors associated with the HIVST kits like variations in age, education, income, or geographical location among men who receive the kits compared to those who don’t. This could help provide insights into which subgroups of men more likely benefit from this intervention and guide targeted strategies for improvement; the detailed nature our study did not do, and lastly the study enrolled male participants who were willing to come to the facility when we invited them, a sense that these participants were motivated and could easily test for HIV thus affecting generalizability.

However, a strength of our study was the ability to reach to men and get first-hand information about the oral HIVST kits they receive through secondary delivery strategy especially since there has not been any follow-up since its implementation in ANC clinics across the country. The findings from our study are important to inform practice, policy, and the Ministry of Health in designing male engagement strategies regarding oral HIVST services.

## Conclusion

HIVST kits delivered through pregnant women reached a high proportion 68.4% men who received the HIVST kits, and uptake was at 82.7% some of whom had never tested to know their HIV status. Factors associated with a high uptake were access to HIVST Information, Education and Communication, being reached at home, and being aware of the partner’s HIV status. The facilitators for use were being convenient and easy to use, fear of negative relationship consequences like breakup, knowing each other’s HIV status as a couple, while barriers like lack of trust in the kit and fear of obtaining incorrect results, an inclination that a woman’s HIV status is the same as theirs affected oral HIVST uptake. Secondary delivery of oral HIVST offers an advantage over the standard facility HIV testing. If scaled up, can contribute to closing the gap towards the first 95 UNAIDS track target; hence having more men tested, given low HIV testing coverage in this population. We recommend integrating the secondary distribution of HIVST kits at all departments in the Hospital in alignment with policies to promote male engagement primarily through women seeking healthcare, provide accurate Information, education, and awareness about HIVST through various channels, social marketing and local media channels promoting HIVST to reach a wider population.

## Figures and Tables

**Figure 1:**
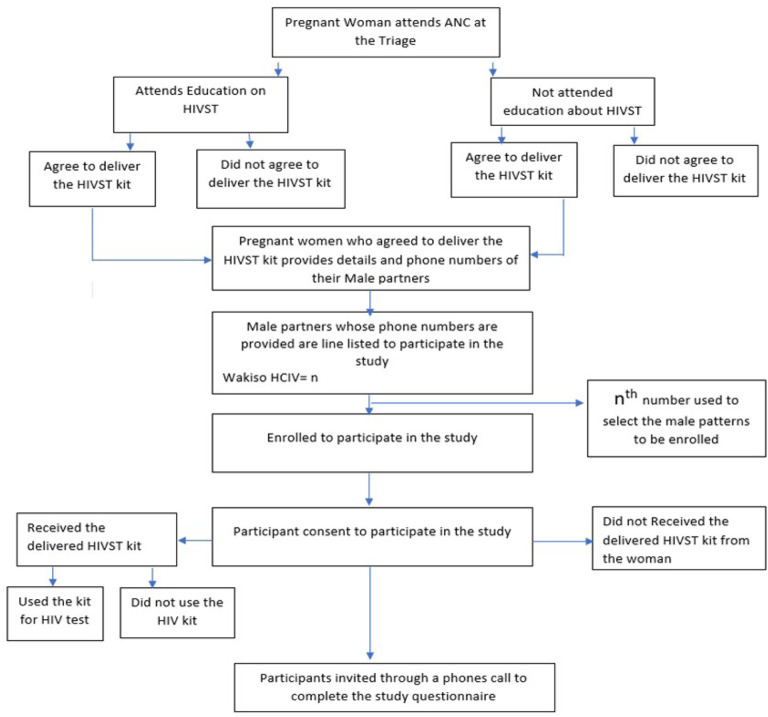
Selection for partners to pregnant women attending ANC at Wakiso HCIV

**Figure 3:**
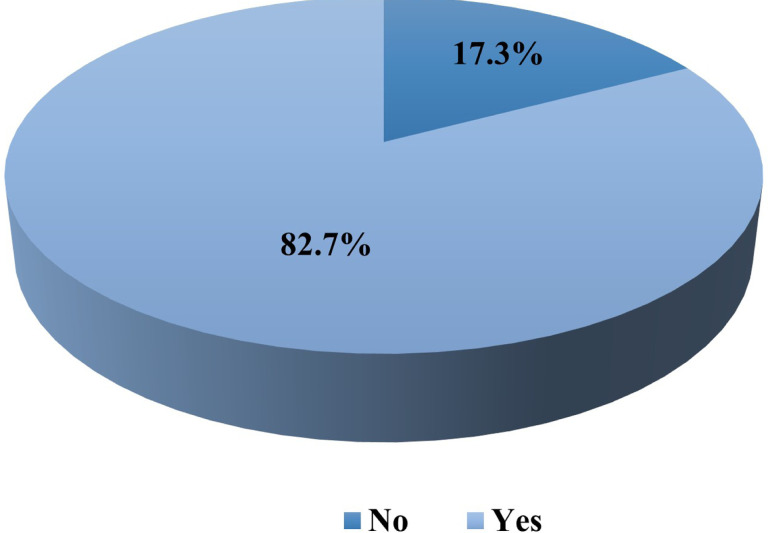
Uptake of HIV oral self-testing among partners of pregnant women.

**Table 1:** Social demographic characteristics of participants

Variable (Categories)		Frequency (n = 380)	Percentage (%)	
Mean Age of Participants (SD) 30.1(6.2)				
Level of income per day Mean (SD) 18554 (29239)				
**Age**				
15–24		60	15.8	
25–34		239	62.9	
Above 35		81	21.3	
**Education level**				
Primary		57	15	
Secondary		252	66.9	
Tertiary		71	18.7	
**Marital status**				
Married		205	53.9	
Cohabiting		175	46	
**Men with more than one spouse**				
I have one spouse		367	96.6	
I have more than one spouse		17	3.4	
**Number of sexual partners**				
I don’t have a sexual partner		173	45.5	
I have at least one sexual partner		207	54.5	
**Place of residence**				
Semi-Urban		72	18.9	
Urban		308	81.1	
**Living with the partner**				
Living with partner		339	89.2	
Staying elsewhere		41	10.8	
**Employment**				
Informal employment		212	55.8	
Formal Employment		168	44.2	
**Religion**					
Anglican	80			21.1	
Catholic	144			37.9	
Muslim	94			24.7	
Other religion	62			16.3	

**Table 2:** Factors associated with uptake of oral HIV Self-Testing among partners of pregnant women

Variable			Uptake HIVST							
			No N = 45		Yes N = 215	Unadjusted CPR(95%CI) P-Value	Adjusted CPR (95%CI) P- Value			
**Age**										
15–24			8(17.8)		38(17.7)	I				
25–34			27(60)		137(63.7)	1.01[0.94–1.08]				
Above 35			10(22.2)		40(18.6)	0.99[0.90–1.07]				
**Education**										
Primary			7(15.6)		34(15.8)	I				
Secondary Education			30(66.7)		144(66.9)	0.99[0.93–1.07]				
Tertiary			8(17.8)		37(17.2)	0.99[0.91–1.09]				
**Marital status**										
Cohabiting			29(64.4)		99(46.1)	I				
Married			16(35.6)		116(53.9)	1.8[1.01–1.11]**				
**Men with more than one spouse**										
I have one spouse			41(91.1)		207(96.3)	I				
I have more than one spouse			4(8.9)		8(3.7)	0.91[0.77–1.07]				
**Number of Sexual Partners**										
I don’t have a sexual partner			23(51.1)		73(45)	I				
I have at least one sexual partner			22(48.9)		118(54.9)	1.02[0.97–1.07]				
**Living with the partner**										
Staying elsewhere			6(13.3)		7(3.3)	I				
Staying with partner			39(86.7)		208(96.7)	1.19[1.00–1.4]*				
**Employment**										
Informal employment			25(55.6)		119(55.4)	I				
Formal Employment			20(44.4)		96(44.7)	1.00[0.95–1.05]				
**Age**											
**Religion**											
Anglican	12(26.7)				45(20.9)	I					
Catholic	15(33.3)				75(34.9)	1.02[0.95–1.10]					
Muslim	9(20.0)				59(27.4)	1.04[0.96–1.12]					
Other religion	9(20.0)				36(16.7)	1.01[0.92–1.09]					
**Received IEC and Education before use of HIVST**											
No	38(84.4)				7(3.3)	I				I	
Yes	7(15.7)				208(96.7)	1.70[1.55–1.87]***				1.64[1.48–1.82] ***	
**Source of information**											
Friends	8(17.8)				29(13.5)	I					
Radio	24(53.3)				116(53.9)	1.03[0.94–1.11]					
Television	13(28.9)				70(32.6)	1.03[095–1.12]					
**Pre-HIV phone counselling**											
No	45(100)				199(92.6)	I					
Yes	0(0)				16(7.4)	1.10[1.07–1.13]***					
**Place of HIV self-testing**											
Facility	43(97.7)				96(44.7)	I				I	
Self-testing	1(2.3)				119(55.4)	1.18[1.12–1.23]***				1.04[1.01–1.08] **	
**Spouses’ age**											
15–24	24(53.3)				108(50.2)	I					
25–34	17(37.8)				98(45.6)	1.02[0.97–1.07]					
Above 35years	4(8.9)				9(4.2)	0.93[0.79–1.08]					
**Age**											
**Spouses’ Education Level**											
Primary Education				12(26.7)	38(17.7)	I					
Secondary education				31(68.9)	150(69.8)	1.04[0.96–1.12]					
Tertiary Education				2(4.4)	27(12.6)	1.09[1.01–19]**					
**Aware of the woman’s HIV status**											
No				11(24.4)	21(9.8)	I					
Yes				34(75.6)	194(90.2)	1.12[1.01–1.24]**		1.04[0.99– 1.09] *			
**Level of Communication with the spouse**											
Regularly				41(91.1)	211(98.1)	I					
Unregular				4(8.9)	4(1.86)	0.82[0.65–1.03]					
**Awareness of HIV status before HIVST**											
No				5(11.1)	3(1.4)	I					
Yes				40(88.9)	211(98.6)	1.34[1.05–1.71]**					
**Number of other sexual partners**											
I don’t have any other sexual partner besides my wife				23(51.1)	97(45.1)	I					
I have at least one Sexual partner				22(48.9)	118(54.9)	1.01[0.97–1.072]					
**Number of children**											
None				10(22.2)	33(15.4)	I					
One child				12(26.7)	82(38.1)	1.06[0.98–1.15]					
More than one child				23(51.1)	100(46.5)	1.03[0.95–1.11					
**Number of Antenatal visits**											
**Age**											
At least 3 ANC		26(57.8)			115(53.5)	I					
More-than 3 ANC		19(42.2)			100(46.5)	1.01[0.96–1.07]					
**Number of spouses**											
Only one		41(91.1)			207(96.3)	I					
More than one		4(8.9)			8(3.7)	0.91[077–1.07]					
**Perceived risk of contracting HIV**											
High		44(97.8)			189(87.9)	I					
Low risk		1(2.2)			26(12.1)	1.08[1.04–1.13]***					

P values (level of significance):

* <0.05;

** <0.01;

*** <0.001

## Data Availability

Data is available upon request from the corresponding author

## Abbreviations

HIVST: HIV Self-Testing
ANC: Antenatal care
IEC: Information Education and Communication
MOH: Ministry of Education
HTS: HIV Testing Services
HCIV: Health Centre IV
